# Barriers and enablers to kangaroo mother care prior to stability from perspectives of Gambian health workers: A qualitative study

**DOI:** 10.3389/fped.2022.966904

**Published:** 2022-08-26

**Authors:** Ying Chun Cho, Abdou Gai, Brahima A. Diallo, Ahmadou Lamin Samateh, Joy E. Lawn, Melisa Martinez-Alvarez, Helen Brotherton

**Affiliations:** ^1^Faculty of Epidemiology and Population Health, London School of Hygiene and Tropical Medicine (LSHTM), London, United Kingdom; ^2^MRC Unit the Gambia at LSHTM, Fajara, Gambia; ^3^Pediatric Department, Edward Francis Small Teaching Hospital, Banjul, Gambia; ^4^Ministry of Health, Gambian Government, Banjul, Gambia; ^5^Faculty of Public Health and Policy, London School of Hygiene and Tropical Medicine (LSHTM), London, United Kingdom

**Keywords:** newborn, premature (babies), skin-to-skin care, Kangaroo mother care (KMC), Kangaroo care (KC), Kangaroo method, Health care worker (HCW), qualitative study

## Abstract

**Aims:**

Kangaroo mother care (KMC) is an evidence-based intervention recommended for stable newborns <2,000 g. Recent trials have investigated survival benefits of earlier initiation of KMC, including prior to stability, with WHO's iKMC trial showing 25% relative risk reduction for mortality of neonates 1–1.8 kg at tertiary Indian and African neonatal units (NNU). However, evidence is lacking about how to safely deliver this intervention to the most vulnerable neonates in resource limited settings (RLS). Our study aimed to understand barriers and enablers for early KMC prior to stability from perspectives of neonatal health care workers (HCW) in a high neonatal mortality RLS.

**Methods:**

This qualitative study was conducted at Edward Francis Small Teaching Hospital (EFSTH), the main neonatal referral unit in The Gambia. It was ancillary study to the eKMC clinical trial. Ten semi-structured interviews were conducted with all neonatal HCW cadres (4 nurses; 1 nurse attendant; 5 doctors; all Gambian). Study participants were purposively selected, and saturation was reached. Thematic analysis was conducted using Atun's conceptual framework for evaluation of new health interventions with methods to ensure data reliability and trustworthiness.

**Results:**

HCW's perceptions of early KMC prior to stability included recognition of potential benefits as well as uncertainty about effectiveness and safety. Barriers included: Unavailability of mothers during early neonatal unit admission; safety concerns with concomitant intravenous fluids and impact on infection prevention control; insufficient beds, space, WASH facilities and staffing; and lack of privacy and respectful care. Enablers included: Education of HCW with knowledge transfer to KMC providers; paternal and community sensitization and peer-to-peer support.

**Conclusions:**

Addressing health systems limitations for delivery of KMC prior to stability is foundational with linkage to comprehensive HCW and KMC provider education about effectiveness, safe delivery and monitoring. Further context specific research into safe and respectful implementation is required from varied settings and should include perceptions of all stakeholders, especially if there is a shift in global policy toward KMC for all small vulnerable newborns.

## Introduction

Complications of preterm birth (<37 weeks' gestation) are the single most common direct cause of death for children under −5 (16%) and for neonates (34%), resulting in 1.1 million neonatal deaths/year (1), with low birthweight (LBW) (<2,500 g) an additional major contributor to neonatal mortality (2, 3). Small, vulnerable newborns are born disproportionately in resource limited settings (RLS) with an estimated 72% of LBW (4) and 81% of preterm neonates (5) born in sub-Saharan Africa (SSA) and Asia. Globally, 30 million neonates require hospital based care every year (6), especially for management of complications of prematurity. Over the last 3 years there has been increasing global recognition and focus on the importance of high quality, facility-based small and sick newborn care (SSNC) in ending preventable neonatal deaths (6, 7). This is urgently required to reach the United Nations Sustainable Development Goal target 3.2 of reducing neonatal mortality to ≤12/1,000 live births by 2030 (8). Despite building momentum and a shift in global policy, critical implementation gaps still exist for SSNC, of which kangaroo mother care (KMC) is central.

KMC was developed in 1978 in South America (9). as a family centered package of care comprising prolonged skin-to-skin contact between newborn and mother or other family member [KMC provider], promotion of exclusive breastfeeding and early facility discharge with close follow-up (10). WHO currently recommends KMC as standard care for stable newborns less than or equal to 2 kg with moderate quality evidence that KMC reduces mortality by 40% compared to conventional incubator care (11). However, nearly half of all neonatal deaths occur during the first 24 h after delivery (12), typically prior to stabilization and in settings lacking neonatal intensive care facilities. Hence, evaluating survival benefits of KMC prior to stabilization was a research priority (11, 13) with several recent or ongoing trials in SSA and India (14–16). The WHO multi-center iKMC trial reported 25% relative reduction in mortality for neonates between 1 kg and 1.8 kg with immediate (median 1.3 h of age) and prolonged (median 16.9 h/day) skin-to-skin contact alongside other SSNC such as bubble continuous positive airway pressure (bCPAP), ventilation and parenteral fluids (15). The iKMC trial findings signal a paradigm shift in SSNC with emphasis on reducing mother-baby separation to promote survival. However, the pragmatic eKMC trial at a more resource limited neonatal unit (NNU) in The Gambia highlighted the challenges of delivering prolonged KMC with substantially lower intervention fidelity (median 6.7 h/d) (14).

Extensive implementation evidence exists for facility-based KMC with stable newborns (17–20) but there is a critical evidence gap for early use with unstable newborns who are a more vulnerable population with specific medical needs. Hence, although there may be overlap, barriers and enablers for KMC prior to stability cannot be assumed to be the same, especially for resource limited settings in which intensive care monitoring and robust health systems may not be available. To date, there is only one small qualitative study from SSA exploring HCW perceptions toward KMC prior to stability, which reported overall acceptability of the intervention to Ugandan HCW (21). There is a paucity of evidence from other RLS with some limited insights into KMC provision on an Iranian maternity unit (22) and feasibility data for ventilated neonates receiving KMC on an Indian NNU (23). HCWs are fundamental for facility-based KMC implementation (20, 24) especially to educate and support KMC providers in delivering KMC. Understanding context-specific HCW perceptions toward provision of KMC to a higher risk, unstable neonatal population is critical for policy and programmatic planning to enable safe and rapid roll-out of this potentially life-saving intervention.

This qualitative study aimed to understand the perceptions of neonatal HCWs toward KMC in unstable neonates <2 kg in a resource limited, high mortality West African hospital setting with exploration of the barriers and enablers influencing early and prolonged KMC delivery.

## Materials and methods

### Study design and context

A qualitative case study design was used to understand lived experiences of HCW, with data collected using a semi-structured interview guide. This study was ancillary to the eKMC trial, a randomized controlled trial investigating the survival and clinical impact of KMC started within 24 h of NNU admission with mild-moderately unstable neonates <2 kg (14).

### Study setting

The study was conducted at Edward Francis Small Teaching Hospital (EFSTH) in The Gambia, a low-income country in West Africa, ranked 173 out of 187 on the Human Development Index in 2018 (25). At time of study onset the NMR was 28 deaths/1,000 live births with an estimated 12% of all Gambian newborns born preterm and 17% born LBW (1).

Edward Francis Small Teaching Hospital (EFSTH) was the only teaching hospital and neonatal referral unit in The Gambia. The NNU admits newborns born at the EFSTH maternity ward as well as those born elsewhere, including home deliveries. Approximately 1,400 neonates are admitted annually with case fatality rates 34% for all newborns and 48% for neonates <2 kg (2010–2014). (26) WHO level 2+ care was provided at the time of study onset, with non-servo controlled incubators (5 functioning), radiant heaters (3), cots (24), bubble CPAP (bCPAP) (3), oxygen *via* concentrators (2), 1 functioning pulse oximeter and burettes for IV fluid administration with availability of 1 fluid pump for blood transfusions. Mechanical ventilation, surfactant, umbilical catheterization and parenteral nutrition were not available. KMC for stable newborns was implemented as standard care in 2017, provided on an 8-bed KMC unit adjacent to the NNU and provided to neonates <2 kg with normal cardiorespiratory status and not requiring oxygen or IV fluid therapy for whom a willing KMC provider was available. KMC prior to stability was introduced in May 2018 as part of eKMC trial implementation with two adult-sized beds provided on the NNU for KMC providers (mothers or other relatives) to provide skin-to-skin contact to trial participants (newborns) alongside other necessary treatments. Alterations to patient flow, reconfiguration of areas within the NNU and provision of additional electric points and an oxygen concentrator were also required for provision of KMC prior to stability. One or two trained nurses and one nurse attendant worked per shift with one research nurse per shift also contributing to clinical care, support of KMC providers and performance of all KMC duration monitoring activities. Medical cover was provided by one consultant neonatologist, one medical officer and two house officers per 24 h shift. KMC was provided to mild-moderately unstable neonates during the study period, as per eKMC trial definitions which were based on cardio-respiratory parameters such as heart rate, respiratory rate and oxygen saturation (SpO_2_) and whether oxygen was required. Neonates receiving bCPAP or who were hypoxic (SpO2 <88%) and/or needing cardio-pulmonary resuscitation were classed as being severely unstable and did not receive KMC (27).

### Study population and sampling

The intended study population was HCW with experience of delivering early KMC prior to stability. This included nursing staff (university trained nurses and nurse attendants) and doctors of varying seniority. Purposive sampling was used to select participants from different roles and seniority levels in order to achieve a range of perspectives representative of neonatal HCW (28). Participants were recruited until thematic saturation was reached (29, 30).

### Study procedures

#### Informed consent and recruitment

Potential participants were approached directly by the lead researcher (YCC) and invited to participate by letter. Written informed consent was then obtained in English language, which all participants were fluent in.

#### Data collection and management

Data was collected during July-August 2018. Interviews were conducted in English using a semi-structured interview guide (Supplementary material 1) in non-clinical rooms at EFSTH at the participants' convenience when they were not on clinical duty. Interviews were recorded on an Olympus WS-853 digital voice recorder. The interviewer was a trained non-Gambian female clinician (YCC), supervised by an experienced qualitative researcher (MMA). She played no role in clinical care or the eKMC trial, although was also supervised by the eKMC lead investigator (HB). The interview duration ranged from 50 to 89 min (average 61 min). The researchers were cognizant of potential social desirability bias, due to the study being embedded in the eKMC trial and recognized that participants may be reluctant to voice negative perceptions of the intervention. To mitigate this the interviewer (YCC) did not engage in any eKMC trial related activities. Internal validity was maintained by one interviewer conducting interviews. Debriefs with a more senior researcher (HB) were done after the first five interviews and included reflexive observations.

Audio-recordings of the interviews were transferred to a password-protected computer after each interview and transcribed verbatim by the interviewer to ensure consistency and dependability (31). Eight consecutive transcriptions were checked for accuracy by two independent English native speakers. Confidentiality was maintained by use of unique identification codes used in all study logs and transcripts.

### Analysis

**Figure 1:**
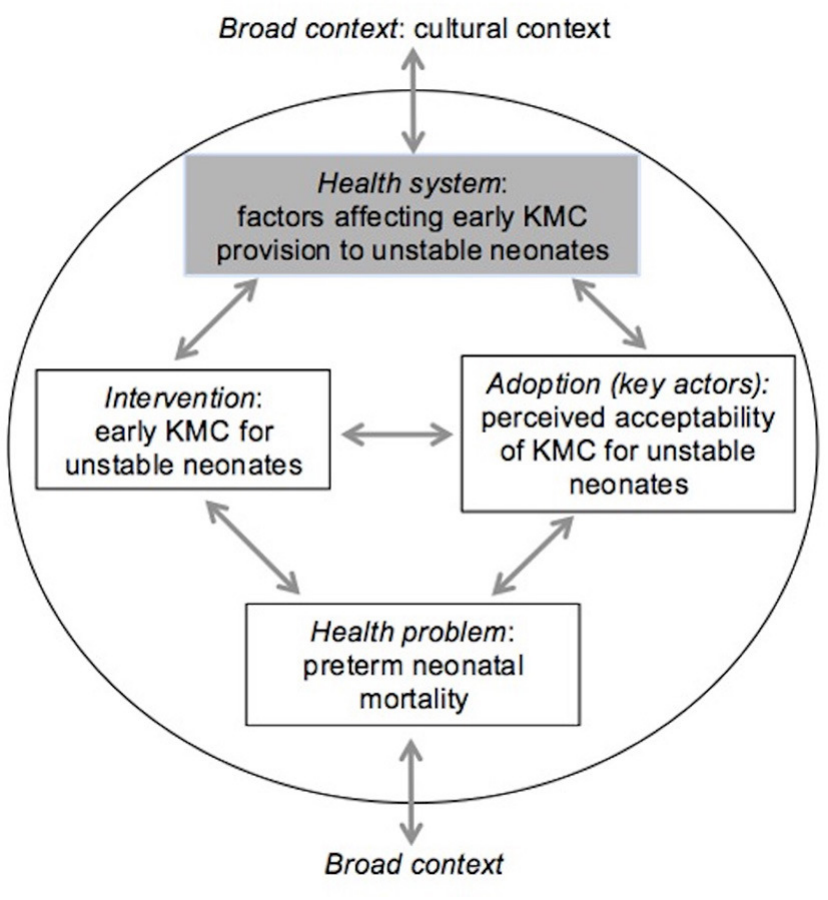
Framework for integration of new health interventions, with relevance for KMC prior to stability adapted from (33).

Data were analyzed using thematic analysis, as outlined by Braun and Clarke (32) using Atun's conceptual framework intended to evaluate the integration of new health interventions (33). This framework has previously been applied in a systematic review of evidence for KMC implementation for stable newborns (34). The framework contains multiple dimensions corresponding to important health system functions: (1) *problem*–health problem targeted by the intervention, (2) *intervention*–including its definition and attributes, (3) *adoption system*–involving multiple key actors and context within which they operate, (4) *health system* and (5) *broader context* (Figure 1). As the problem of small vulnerable newborns has been extensively described elsewhere in the literature this was not included in the analysis.

Transcripts were deductively coded line-by-line by the interviewer (YCC), with concepts labeled in relation to the study aim with a word or a phrase. NVivo 11 (QSR International, Cambridge, MA) was used to assist coding and theme identification. Codes were aggregated into sub-themes based on similarity of their relation to each other. The sub-themes were then deductively grouped and merged into themes by applying the key components of the framework, which thereafter were reviewed and refined to ensure relevance to the study aim. Pseudonymized quotes were selected to reflect the themes and sub-themes. This article was prepared according to Standards for Reporting Qualitative research (SRQR) (Annex II) (35). Ethical approval was granted by the Gambia Government / MRC Joint Ethics Committee, The Gambia (Ref. 1610) and the LSHTM Ethics Committee (Ref. 15357).

## Results

We begin by describing the participant characteristics and then proceed to present the identified themes and sub-themes, grouped as per Atun's conceptual framework, and providing a narrative description of main findings as perceived by Gambian HCWs.

### Participant characteristics

Ten HCW participated: four trained nurses, one nurse attendant and five doctors (Table 1). All participants were Gambian nationals and underwent undergraduate medical or nursing training at Gambian higher educational institutions, apart from one doctor who was trained in Venezuela and the nursing attendant who received vocational training. Six HCWs had previously been trained on care of the small and sick newborn and KMC (2017) and all had been sensitized about the eKMC trial within the preceding 3-months, including provision of early KMC prior to stability.

**Table 1:** Demographic and professional details of health care worker participants.

		**All**	**Nurses**		**Clinicians**	
			**Registered nurse**	**Nurse attendant**	**Medical officer**	**House officer**
	**TOTAL**	**10**	**4**	**1**	**2**	**3**
Sex	Female	5	3	1	0	1
	Male	5	1	0	2	2
Age (years)	≤30	3	1	0	1	1
	31–40	5	2	0	1	2
	41–50	2	1	1	0	0
Newborn care experience (years)	<1	3	1	0	0	3
	1–3	4	1	0	2	0
	>3	3	2	1	0	0

### Themes and perceptions

Multiple factors were identified as being important for the provision of early KMC prior to stability and are presented as barriers and enablers as per the pre-defined themes from Atun's conceptual framework (Table 2).

**Table 2:** Barriers and enablers for delivery of KMC prior to stability, as perceived by Gambian health care workers.

	**Intervention: KMC prior to stability**	**Adoption system: Acceptability of early KMC prior to stability to key stakeholders**		**Interaction with health system**	**Cultural context**
		**Health care workers**	**KMC providers**		
**Barriers**	• Limited understanding of rationale • Uncertainty about effectiveness	• Safety concerns -IV fluid administration -IV cannula dislodgement -KMC wrappers -Infection risk -Gastric tube feeding • Lack of clinical monitoring	• Availability of KMC provider -Illness, post-operative, admitted at another facility -Domestic responsibilities -Prolonged admission • Physical discomfort -Tiredness, back pain -Sleep disturbances -High ambient temperature • Lack of privacy/respectful care • Negative perceptions of KMC • Lack of understanding -Communication barriers	• Health system limitations -Lack of adult beds -Lack of space with risk of overcrowding -Limited WASH and cooking facilities • Staffing shortages	• Exclusion of fathers from newborn care • Socio-cultural norm of carrying newborns on back • KMC as new practice in The Gambia
**Enablers**	• Transfer of knowledge from existing KMC practices • Recognition that early KMC may improve thermal control and stability	• HCW education and experience -HCW prior experience -Continuous education including at antenatal clinics • Methods for safe IV fluid, oxygen and gastric tube feeding • Pulse oximetry monitoring during KMC prior to stability • Task shifting to reduce HCW workload	• Family support -Provide KMC -Emotional support • Education of KMC providers -Benefits and safety of KMC -Compliance with HCWs • Empowerment as main carer -Enhanced bonding -Effects of KMC on newborn • Peer-to-peer support	• Comfortable environment -Distractions (e.g., TV) -Cooking and WASH facilities • Reduced HCW workload • Rationalization of scarce resources • Improved patient flow with earlier discharge	• Prominent role of women during perinatal period • Buy-in from fathers • Community sensitization

*HCW, health care worker; IV, Intravenous; KMC, Kangaroo mother care; NNU, neonatal unit; TV, Television; WASH, Water, Sanitation and Hygiene*..

#### Intervention (early KMC prior to stability)

All participants were aware of KMC as a method of care for small vulnerable newborns with recognition that skin-to-skin contact is a key component. Participants overwhelmingly expressed positive attitudes toward KMC for stable neonates, regardless of their HCW role, seniority or experience. There was good understanding of the benefits of KMC, which participants identified as: breastfeeding promotion; faster weight gain; early discharge and avoidance of infections. Participants recognized the important role of KMC for thermal control, especially if incubators or radiant warmers were unavailable, as “*caregivers are just like radiant warmers”* as the nursing attendant observed. Participants drew on their professional experience of KMC for stable newborns and cited examples of positive outcomes compared to conventional incubator care, as mentioned by one doctor “*I've seen a lot of improvement with the contact of mother and baby…is even more better than putting babies in incubator”* [HCW01].

In contrast, there were divergent perceptions of early KMC prior to stability with no consensus amongst HCWs about acceptability of the intervention. There was limited understanding about the rationale for providing KMC to unstable neonates and half of the participants were hesitant about the intervention, with three junior HCW expressing more doubts and one doctor commenting that “*I think it's best to wait until at least the first 24 to 48 h before we initiate KMC [to the unstable neonates], because those are just critical, and you want to be on top of things”* [HCW05]. More than half the HCW thought that early KMC could prevent hypothermia and two HCW mentioned enhanced neonatal stabilization as a potential benefit. All cadres of HCW expressed uncertainty about the effectiveness of the intervention, with one nurse preferring not to implement early KMC prior to stability unless recommended by global guidelines: “*For the stable ones, it works pretty well, but for the unstable ones, I can't say that it's working… that well”* [HCW04].

Other participants accepted that KMC could be provided alongside other treatments, with extrapolation of existing knowledge of KMC for stable newborns especially regarding benefits of thermal control: “*The temperature is very important to preterm babies, as they can lose [heat] very easily. The skin-to-skin contact is beneficial as well. So, I believe it's part of the treatment, giving other treatment shouldn't stop you from doing KMC.”* [HCW01] Two doctors believed that KMC should be provided for “*as long as the mother can”* [HCW01] although target duration was not mentioned by any HCW. More experienced neonatal HCW expressed more acceptance of the intervention, with an expectation by more senior HCWs that early KMC could be beneficial if proven to be safe.

#### Adoption system (health care workers and KMC providers)

The adoption system refers to the key actors involved in provision of the intervention and their interests, values and interactions (33). As both HCW and KMC providers (mothers and families) are important actors in this complex intervention (36), this theme was sub-divided to enable consideration of factors related to each actor.

#### Factors related to health care workers

Perceptions of KMC prior to stability relating to HCW factors centered on three sub-themes: (1) Importance of HCW education; (2) Safety of KMC alongside other small and sick newborn care interventions; (3) Need for vigilant clinical monitoring of unstable newborns receiving skin-to-skin contact.

##### Importance of HCW education

Education of HCW about the importance and practicalities of early KMC prior to stability was seen as central for successful delivery, for both permanent HCW and rotating junior doctors and nurses. Expanding education activities to include HCW in antenatal clinics was also suggested by two senior nurses to enable prenatal sensitization and facilitation of KMC as soon as possible after delivery: “*If you tell them that since at the antenatal clinic, they will be aware of it…, they will be preparing for it, so it would not be a problem”* [HCW11].

##### Concerns about safety of KMC alongside other small and sick newborn care

Safety of the intervention was an important sub-theme with concerns expressed by all participants, but especially more junior HCW. One nurse expressed concerns that newborns could deteriorate if KMC is started too early. Participants also voiced concerns about providing early KMC in specific high risk clinical scenarios, such as: apnea; respiratory distress; severe jaundice requiring phototherapy; blood transfusion; convulsions or high fever.

Views on the provision of early KMC alongside other SSNC interventions varied according to the type of treatment. KMC given simultaneously with IV fluids was considered feasible but 6 participants, mostly junior HCW, expressed concerns regarding safe IV fluid provision with use of the KMC wrapper. Specific concerns included occlusion of IV lines if KMC wrappers were tied tightly or incorrectly and risk of peripheral venous cannula dislodgement due to flexed position or compression of limbs within the KMC wrapper. One doctor noted that “*They tie [the wrappers] on top of the fluid, so it compressed the vein and even IV fluids, the giving set, it compressed so it stopped”* [HCW02]. Two doctors described experiences in which neonatal dehydration or hypoglycaemia had occurred during KMC provision to unstable neonates, which they attributed to the intervention. Half of the participants reported that IV cannulas had become dislodged during provision of the intervention and raised the possibility that seclusion in the KMC wrapper would delay detection of cannula problems.

Practical solutions to promote safe IV fluid delivery whilst in KMC position were suggested. These included secure placement of cannulas in upper limbs (not lower limbs), positioning of hands outside of the KMC wrapper, education of KMC providers about appropriate wrapper tying and limitation of movements and observing fluid drip rates in-order to promptly identify fluid administration problems.

Several HCW also reported a perceived increased risk of infection associated with the effect of KMC on IV cannula sites, with one doctor observing that “*sometimes skin-to-skin contact, you see mother is sweating and when they sweat, sometimes they get, the cannula got sweat. And those are risk factors of polluting, infections to the line”* [HCW01].

Oxygen provision was not thought to be affected by KMC delivery unless the newborn was severely unwell, but some participants did identify this as a concern for KMC providers. An experienced doctor commented that “*The mothers sometimes are a bit worried, they think it [oxygen] is going on and off when they move up and down, but we explain to them that we secure it correctly and they can receive it fully ”* [HCW08]. A senior nurse identified the importance of securing the oxygen prongs with tape.

Perceptions of the use of nasogastric (NG) tube feeding whilst in KMC position varied, with participants expressing contradictory views about whether KMC aided feeding or exacerbated the risk of milk aspiration from being compressed within the KMC wrapper. Junior HCWs expressed more reluctance regarding NG feeding alongside KMC with one junior doctor stating “*Because they [are] compressed, and the child might regurgitate or vomit when they're being fed in that position”* [HCW05].

##### Need for vigilant clinical monitoring of unstable newborns during KMC

The need for vigilant clinical monitoring of unstable neonates was highlighted by several participants with a general reluctance to place unstable neonates in KMC position without direct observation and continuous pulse oximetry monitoring. Specific concerns included that the KMC wrapper may prevent visual observation of unwell neonates and timely detection of illness, with one doctor commenting that “*You have a critical child who needs a lot of supervision, who needs to be looked at, you need to visibly see these children”* [HCW05].

Four experienced HCWs perceived that KMC providers play an important role in monitoring unstable newborns, with one doctor stating that “*the mom is in contact with the baby 24 h, as soon as something happens, it [she] would notify. So, it's actually better than not doing it early”* [HCW06].

This task shifting of the supervisory role onto KMC providers was valued by several HCW as a way of reducing HCW workload as HCWs were just there to “*monitor and supervise”* [HCW8], as said by one senior doctor. Only one nurse reported increased workload due to attending to KMC providers' needs.

#### KMC providers

Several factors relating to KMC providers were viewed as being important for delivery of early KMC prior to stability, including: (1) Availability of the mother during the early admission period with family support; (2) Physical discomfort, especially during sleep; (3) Importance of privacy during skin-to-skin contact; (4) Education of KMC providers in context of high compliance with HCW authority; and (5) KMC provider empowerment and peer-to-peer support. Each of these factors are considered in turn below:

##### Availability of mother with family support

Maternal unavailability on the NNU was the biggest challenge to delivering early KMC, as perceived by all participants. This was attributed to a variety of reasons including: postpartum illness; admission at another health facility or recovery from cesarean section. One doctor commented that “*When the mother is in post-*cesarean*-section, you're not able to transport the mother from postnatal [ward] to this place [NNU], or especially when the mothers, the babies are far, from other health facilities, you might not get the mother in the first 24 h”* [HCW02].

HCW generally viewed KMC prior to stability as an intervention which had to fit around the KMC providers' other responsibilities, with several reasons identified as limiting the delivery of prolonged KMC. These included domestic responsibilities such as childcare of other children, and lack of access to WASH facilities such as toilets, showers and running water. A senior nurse identified that lack of WASH facilities prevented prolonged continuous early KMC, stating that “*Inside there [is] one toilet, so sometimes we go up to 40 something babies, it's difficult, so some they [KMC providers] would not like to use the toilet, they would go down and find where to take bath”* [HCW09]. Prolonged admission was viewed by half of participants as a hindrance to KMC provider's commitment to KMC due to financial burden on the family to provide food and other necessities. Five participants considered that the quality and quantity of hospital provided food did not meet the mother's needs and reported that many mothers rely on families' donations, with an experienced doctor stating that if the length of stay is too long the KMC provider's families can become “*fed up [with] bringing food”* [HCW08].

Family support from grandmothers or aunties was viewed as essential with one doctor sharing that “*sometimes we bring second help as a way of accompanying them to feel that they're at home…not necessarily doing KMC, but to be with them”* [HCW08]. Female relatives were seen by most HCW as feasible alternative KMC providers if the mother was unavailable, with one nurse reporting that “*We once had a mother, the mother was not feeling very well, so a helper was inside helping her. But some of them, their relatives are not here, they came alone here, that is the problem*” [HCW11]. Relatives were also reported to provide emotional support to mothers. However, two nurses expressed a preference for mothers as KMC providers with one nurse commenting that “*We should know which types of helpers we're taking, if the hygiene is poor or you know that this one is not that active, I think we wait for the mother”* [HCW09]. Frequently changing KMC providers and having many additional KMC providers on the NNU was also seen as an infection prevention control risk.

##### Physical discomfort especially during sleep

Physical discomfort was viewed as the most frequent complaint made by KMC providers. Causes of discomfort were thought to include tiredness and back pain from extended periods carrying newborns in KMC position as well as post-operative pain. The effect of high ambient temperatures was also mentioned as a cause of women's discomfort during KMC.

Sleep deprivation, poor sleep quality and KMC provider concerns about safety of their newborn during sleep were also important barriers for KMC provision, as noted by the nursing attendant: “*They [Mothers] might tell you ‘I'm tired, I need to turn, I cannot turn with my child, it's not safe’, unless you take the child from them, place them on the radiant heater, allow them to sleep”* [HCW10].

##### Lack of privacy during skin-to-skin contact

A lack of privacy on the busy NNU was perceived by two female nurses as a key factor limiting the acceptability of KMC to mothers and female relatives, with women reluctant to expose their bodies during skin-to-skin contact, as one nurse observed: “*They [Mothers] said ‘No, no, no, I cannot undress myself here inside this public place. They rather, just [let the baby] stay inside the incubators”* [HCW09]. The lack of curtains or screens was identified by one doctor as a possible factor in fathers’ infrequent visits to the neonatal and KMC units: “*If one of the fathers has to come to the KMC ward…because the mothers have to be exposed for the skin-to-skin [contact], so [they] could get very awkward and really uncomfortable. It's a shared ward”* [HCW05.] Gowns were provided for KMC providers, but HCW considered that the hot climate precluded women from using them in addition to the KMC wrapper.

##### KMC provider education in context of high compliance with HCW authority

KMC providers' negative perceptions about KMC prior to stability and lack of understanding were highlighted as important barriers for maternal and family buy-in to the intervention. The capacity of mothers to understand was seen as foundational, with two HCW's commenting that multiple ethnic languages posed a communication challenge and one nurse described some mothers as being “*difficult”* and requiring repeated explanations.

Experienced HCW of all cadres recognized the importance of educating KMC providers about the benefits of KMC prior to stability and how to safely deliver the intervention, with the nurse attendant suggesting that “*There should be everyday, or every 2 days, giving health talk to the mothers for them to know what's the neonatal KMC, because we said in our own languages, so they will understand it more”* [HCW10]. Three participants stated that mothers and other family members would accept intravenous (IV) fluids with KMC if they understood that both treatments were beneficial, as all they [KMC providers] want is “*to see their babies getting well*”.

There was general recognition that parents want the best for their newborns and frequent statements about mothers being compliant with HCW's instructions. Many of the participants considered their own role in educating mothers and relatives, with a frequently expressed view from both nurses and clinicians that HCWs garner a high level of respect and can exert a positive influence on parental behavior, as described by one doctor “*Even though initially they feel a bit strange, but it depends on the way you explain to them. So, if you, as a health worker, you show the mother something strange, the mother will take it as strange thing. When you explain to mother that it's normal and it's for the benefit of the child. So, they will do it”* [HCW01]. The importance of the HCW hierarchy was also considered to be a factor in maternal and family acceptance of KMC prior to stability. One clinician perceived that KMC provider compliance may be enhanced if KMC is described as a medical treatment versus a nursing method, although this finding was not reported by nursing HCW.

##### Empowerment of KMC providers and peer-to-peer support

Mothers' who directly observed beneficial effects of KMC on their newborns' sleep and clinical status were thought to be more accepting of the intervention. One nurse attendant described her experience being told by a mother that “*my child is now used to this KMC”* [HCW10] because the baby was able to sleep more and cried less whilst in KMC position. Enhanced bonding and attachment between KMC provider and newborn was thought to enable KMC for some women. Three participants perceived that spending more time with their newborn as primary care-giver empowered mothers and was an important enabler for delivery of this intervention, as stated by one doctor: “*They [Mothers] take ownership of the work, they think they're doing the work for themselves… the mothers are very happy with that”* [HCW08].

Peer-to-peer support was also seen as valuable to encourage mothers' uptake of KMC prior to stability with discussion of their experiences in supporting stable newborns and transfer of knowledge for KMC prior to stability. The role of other KMC providers was described by four participants as explaining or translating HCW instructions as well as reminding about feeding times, maintaining hygiene and sharing knowledge about comfortable and safe KMC provision. One senior doctor shared that “*…always one mother who is experienced than the rest, and she takes the lead and takes the charge and teach them”* [HCW06].

#### Interaction with health system

Participants highlighted several health system limitations affecting delivery of early KMC prior to stability, including provision of a comfortable environment, availability of beds and space, access to cooking or WASH facilities and HCW availability.

Several HCW viewed the environment as important, including access to cooking facilities and, as suggested by one nurse, distractions such as a television to reduce the tedium of staying in hospital for prolonged periods. A senior doctor stated that “*If there's an environment that makes them to feel like home… they have a place where they can cook, they get what they want to cook… will be a great encouragement for mothers to stay as long as they could for the interest of their babies”* [HCW01].

Limited availability of beds for KMC providers on the NNU was also viewed as a key barrier to delivery of early KMC prior to stability by half of the participants, with recognition that beds were required for KMC providers' comfort. A senior nurse stated that “*Because early KMC you need beds, and we don't have space for beds… right now it's only two beds, so if we want to all that unstable babies to start KMC, we cannot”* [HCW09]. Participants also considered the capacity of the NNU space to accommodate additional beds and KMC providers, with concerns about over-crowding leading to higher infection risk. Two nurses were in favor of allowing only mothers onto the NNU due to space limitations and to prevent infection. Establishment of a special ward for provision of early KMC was proposed with views expressed that newborns receiving KMC should be cohorted in a separate area to outborn or potentially infectious neonates in an effort to reduce infection risk for the vulnerable small newborns. The nurse attendant suggested “*[Open up] a special ward with the special nurse,… because different babies are here, not from only the labor ward [in this hospital] but referred from [other] facilities,…so infection might come very easy”* [HCW10].

Limited access to basic water, sanitation and hygiene (WASH) facilities, such as toilets, showers and running water for hand hygiene was seen as interrupting prolonged KMC for both stable and unstable newborns by all participants. Staffing limitations were also thought to influence the clinical monitoring and management of unstable neonates receiving KMC with the nursing attendant reporting that “*The fluids get stopped overnight, when most staff, we have few staff on the ground and sometimes the mothers also feel sleepy, they cannot observe that the fluid is not flowing at all, so when you come in the morning, this child is already hypoglycemic”* [HCW10]. Three doctors also raised the risks during the night shift when HCW staffing levels are low and KMC providers are sleeping.

Participants identified several benefits for the health system, including reduced HCW workload due to enhanced monitoring by KMC providers, rationalization of scarce resources as “*you just need the mother and child, and that's it”*[HCW05] and improved patient flow through the neonatal unit via enhanced stabilization leading to earlier discharge.

#### Cultural context for early KMC prior to stability

The importance of gender roles within newborn care decision making was highlighted by all HCWs, with identification of the peripartum period as being dominated by women. Exclusion of fathers from pregnancy, childbirth and child-care was mentioned by four HCWs, with one junior doctor stating that “*it is believed that the whole period, the whole puerperium, the childbirth, the pregnancy is for women, it is not for men, and men are totally excluded from it”* [HCW05]. However, three male participants believed that the father held the ultimate authority within the household and that paternal buy-in and encouragement were necessary for women to engage with the intervention. A senior male nurse commented that “*The mother might not like something, but with the interference of the husband… she will tend to do it [KMC]”* [HCW04].

Community understanding and acceptance of KMC was also viewed by four HCW as being essential so that mothers would not “*feel strange”* for front-carrying their newborns instead of back-carrying, which is the accepted socio-cultural norm. One senior nurse commented that “*Some of them (mothers) think that it's not the safer method, because of the taboo. So, some of them think that the baby should be always at the back, not in the front, that's our belief here”* [HCW04]. Community sensitization about KMC as a way of caring for small babies and, especially, prior to stability, was suggested as a strategy to enhance individual buy-in, with recognition that KMC is a relatively new practice in The Gambia.

## Discussion

This qualitative study presents novel insights for delivery of KMC prior to stability on a West African resource limited NNU with value for regional roll out if global policy change occurs. Important barriers included: Maternal unavailability early in NNU admission; Physical discomfort of the KMC provider; Perceptions around unsafe delivery alongside other small and sick newborn care interventions; and health system infrastructure and resource limitations including lack of space, beds, WASH facilities and staffing. Key enablers included: HCW training with consideration of “task-shifting” onto KMC providers; Provision of respectful care for KMC providers; Sensitization of mothers, fathers, families, communities; and peer-to-peer support.

Unavailability of mothers during early NNU admission was a foundational barrier with multiple reasons identified why mothers were not available at our study site. Over one-fifth of newborns in the eKMC trial intervention group were born by Cesarean section, with 52% of the trial cohort born at another health facility (14) and geographical separation of the EFSTH neonatal and maternity units. Understanding the precise reasons for maternal unavailability is important to help facilitate earlier maternal presence and will likely vary between health facilities. Involving other family members as alternative KMC providers may mitigate early maternal unavailability, as identified by HCW in this study and as per the eKMC trial in which 46% of the intervention arm received first KMC from a female relative (14). Female relatives are willing to support mothers in this role (36) and are influential newborn care decision makers in many African communities (37, 38). Hence, involving female relatives, especially elders, in policy and programs may assist with delivery of this complex intervention.

Physical discomfort experienced by KMC providers, especially during sleep, was seen as a key barrier for prolonged KMC prior to stability. Sleeping supine or upright whilst in KMC position is a recognized barrier to KMC practice in both LMIC (20, 34) and HIC settings (39) and resources such as pillows, washable mattresses and secure, comfortable KMC wrappers can relieve KMC provider discomfort (20, 34). Mothers are recovering from child birth at time of early KMC initiation, therefore issues pertaining to mothers' physical health and sleep quality likely play a greater role in delivery of early KMC compared to KMC later in the postnatal period. An effective strategy during the iKMC trial was the establishment of “mother-neonatal ICUs”, with joint maternal and neonatal management in a shared space with input from specialist neonatal and maternity HCWs (15). Further insights into implementation of “mother-neonatal ICU's” from the iKMC trial is awaited, especially costing tools and data to inform infection prevention control planning, which was a specific concern expressed by our participants with regards to overcrowding and presence of multiple family members on busy NNUs.

**Figure 2:**
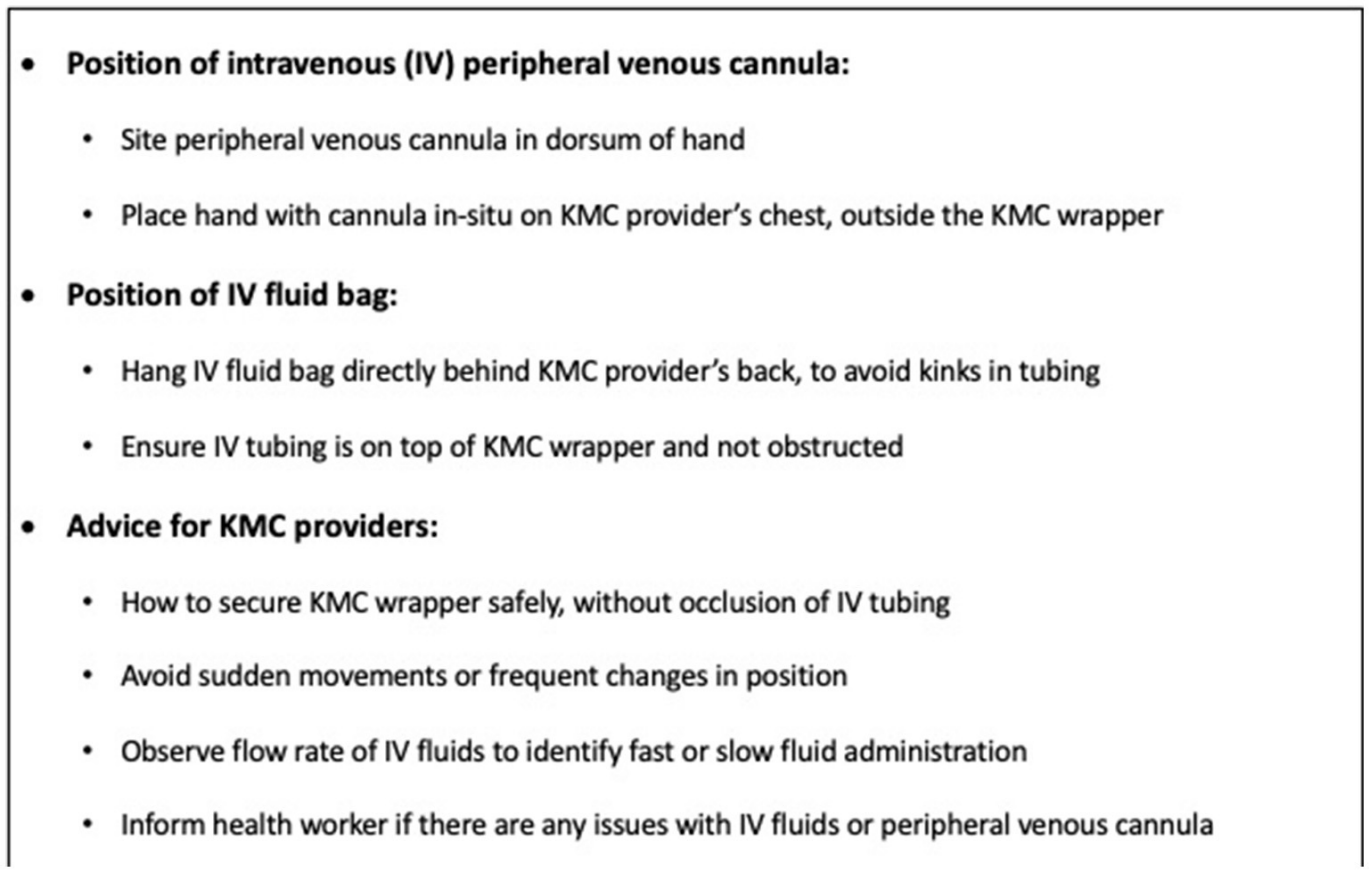
Recommendations to enhance safety of intravenous fluid administration whilst in KMC position.

Safety was a key concern for our participants, especially regarding safe IV fluid administration whilst in KMC position. This contrasts with Ugandan HCW, who had no safety concerns themselves but reported that Ugandan mothers were concerned about IV tubing dislodgement during KMC (21). The KMC wrapper was thought to impact on safe IV fluid provision by Gambian HCW. There is limited data comparing safety of different KMC wrapper types for unstable populations in RLS (40) and this is essential to know prior to widescale implementation of KMC prior to stability. We addressed safety concerns in real-time by refining eKMC trial procedures to minimize disruption to IV fluid administration with enhanced education of HCW at the trial site using recommendations from our findings (Figure 2). Providing KMC to unstable newborns alongside oxygen was not perceived as a barrier by Gambian HCW, consistent with findings from elsewhere in Africa (21).

Clinical monitoring during KMC prior to stability was deemed essential by our participants with pulse oximetry especially important at night due to limited staffing and KMC providers' need to sleep. Both Ugandan HCW and mothers also identified the need for pulse oximetry for unstable newborns receiving KMC (21). However, perceptions varied about the effect of KMC position on monitoring, with recognition that whilst KMC can enable earlier detection of problems such as apneoa by KMC providers, there is reduced direct monitoring by HCW due to encasement within the wrapper (21). There is no evidence that providing KMC to unstable newborns in RLS is unsafe (14, 15, 23), but HCW perceptions or doubts about safety may contribute toward HCW reluctance to comply with any future recommendations to expand KMC to an unstable population. This is an important barrier to HCW buy-in and could be mitigated by involvement of HCW in local implementation and focus on safe IV fluid administration.

Lack of adult beds on the NNU and limited space were key health systems barriers for early KMC prior to stability, consistent with existing implementation evidence for KMC from the perspective of KMC providers (21, 34, 41), health workers and health systems (20, 24). We addressed these findings during the eKMC trial by reconfiguring the NNU to allow space for four adult beds with provision of additional electric points and an oxygen concentrator. This required support from hospital administrators as well as additional funds, highlighting the importance of KMC champions at all health system levels (18) especially strong local leadership and adequate financing (20). Understanding the perspectives of administrators toward health systems change to facilitate KMC prior to stability is essential and a current research gap, along with economic evaluations. Provision of adequate WASH facilities close to the NNU is also an important health system requirement for prolonged early KMC, as identified by Gambian HCWs. This would reduce time that KMC providers spend away from their newborns and also promote infection prevention control practices which our participants highlighted as a specific concern.

Limited staffing levels and high workload can preclude KMC provision for both stable (20) and unstable newborns, as we identified. The iKMC trial provided HCWs with responsibility only for supporting KMC providers, which possibly contributed toward the higher intervention duration delivered (median 16.9 h/d) (15). This contrasts with only 6.7 h/d of KMC delivered in our more pragmatic eKMC trial, when research and hospital personnel had multiple clinical responsibilities and a high workload burden with nursing to patient ratios being as low as 1:40 newborns during peak admission periods (14). Feasible strategies are required to overcome this so that HCW can adequately support KMC providers. Task-shifting of appropriate duties to KMC providers with promotion of family integrated care (42) may mitigate this barrier and our findings suggest that this would be acceptable to Gambian HCW.

HCW training about KMC theory and practice, linked to accessible protocols with supportive programmatic supervision is the cornerstone of existing KMC practice (20, 24) and our findings support this strategy for KMC prior to stability. Sharing knowledge within professional and hospital networks with mentorship visits, peer-led workshops (20) and the Communities of Practice model (43) would also aid implementation. Our participants focused mainly on skin-to-skin aspects of KMC with relatively scant mention of exclusive breastfeeding or feeding support. This absence of HCW focus on feeding was also observed by a systematic review synthesizing existing evidence for barriers and enablers of facility-based KMC in SSA (20). This underlines the importance of comprehensive HCW training including emphasis on promotion of exclusive breastfeeding so as to promote knowledge transfer to KMC providers.

Respectful treatment of KMC providers was seen as an important enabler by Gambian HCW, with privacy perceived as fundamental. This is consistent with an Iranian study in which 83% of midwives considered privacy as a key enabler for KMC in the delivery suite (22) and the OMWaNA feasibility study in which both Ugandan mothers and HCW stressed the importance of privacy on busy NNUs (21). Privacy screens can promote KMC acceptance and delivery in HIC settings (44, 45) and should also be promoted in LMIC settings. Providing a comfortable and safe environment for KMC is consistent with WHO standards for respectful maternal and newborn care (46) which also includes respectful communication between HCW and KMC provider (20).

This study adds to the literature by presenting HCW's lived experiences of delivering early KMC to unstable neonates in an African RLS and provides important contextual insights from a pragmatic research setting with value for future research, practice and implementation. We identified contrasting perceptions of HCW toward KMC prior to stability with doubts expressed about intervention effectiveness. However, interpretation of this is limited, as data was collected during the early phase of the eKMC trial, prior to publication of iKMC trial results and, hence, the effectiveness of the intervention was not known at time of study onset. Our methods were robust with utilization of a relevant conceptual framework (33) and we made efforts to ensure data reliability and trustworthiness such as quality checking of transcriptions and maintenance of internal validity.

Limitations included data collection by a non-Gambian, with potential for misinterpretation of culturally specific findings. Interviews were conducted in English which may not have been the participant's preferred language, despite high fluency levels, and nuanced descriptions may have been missed. Despite efforts to reduce social desirability bias, association with the eKMC trial and the locally well-regarded research institution (MRCG at LSHTM) may have biased our findings. However, as participants expressed mixed perceptions of the intervention with multiple concerns and no overwhelmingly positive HCW perceptions, social desirability bias is unlikely to have played a prominent role. The small sample size is acknowledged but as all HCW cadres were included and thematic saturation was reached, we are satisfied that our findings represent those of Gambian neonatal HCW with experience of supporting KMC prior to stability. Extrapolation to other settings should be done with caution due to the specific factors relating to setting, population and research context. We present a HCW centered perspective and findings related to KMC providers' experiences should be interpreted with caution, as we did not specifically explore the interaction of HCW and KMC providers nor include KMC providers' own perceptions.

Understanding views of other key stakeholders involved in KMC is urgently needed prior to wide scale roll out or policy change. This should include mothers and fathers, the extended family especially female relatives, community women's groups and religious leaders. Understanding the attitudes and priorities of hospital administrators, policy makers and program managers is also critical with economic evaluations at health system, family and societal levels and from varying RLS settings vital for health service planning. Implementation insights from other trials are awaited and will add to this evidence base, with anticipated high value from the OMWaNA trial economic evaluation (16).

## Conclusion

Early KMC prior to stability is a life-saving intervention for vulnerable newborns and signals a paradigm shift in family centered small and sick newborn care. Gambian HCW expressed contrasting views toward this intervention with uncertainty about effectiveness and safety concerns especially regarding concomitant IV fluid administration and impact on infection prevention control. Health systems limitations (beds, space, WASH, staffing), unavailability of mothers during the early neonatal period and need for respectful care were deemed as important factors restricting delivery to unstable newborns on this resource limited NNU. HCW training leading to KMC provider education, family and peer-to-peer support and sensitization of fathers and communities were identified as potential enablers. Further context-specific implementation research from varied RLS and all stakeholders is urgently required for health service planning of safe and respectful operationalization of early KMC prior to stability, if this potentially life-saving intervention is recommended by global policy.

## Data availability statement

The raw data supporting the conclusions of this article will be made available by the authors upon request to the corresponding author.

## Ethics statement

The studies involving human participants were reviewed and approved by LSHTM Observational Ethics Committee, LSHTM and the Gambia Government / MRC Joint Ethics Committee, The Gambia. The patients/participants provided their written informed consent to participate in this study. Written informed consent was obtained from the individual(s) for the publication of any potentially identifiable images or data included in this article.

## Author contributions

YCC, HB and JEL conceptualized the study. YCC developed the study guides and obtained regulatory approvals with input from MM-A and HB. YCC collected and analyzed the data with support from HB and MM-A. YCC prepared the first draft of the manuscript with substantial input from HB and technical advice from MM-A and BD. All authors contributed towards and approved the final version.

## Funding

YCC received funding from LSHTM Travel Award fund and the study was embedded within a Wellcome Trust Research Training Fellowship awarded to HB (Ref: 200116/Z/15/Z).

## Conflict of interest

The authors declare that the research was conducted in the absence of any commercial or financial relationships that could be construed as a potential conflict of interest.

## Publisher's note

All claims expressed in this article are solely those of the authors and do not necessarily represent those of their affiliated organizations, or those of the publisher, the editors and the reviewers. Any product that may be evaluated in this article, or claim that may be made by its manufacturer, is not guaranteed or endorsed by the publisher.

## Abbreviations

bCPAP: Bubble continuous positive airway pressure
EFSTH: Edward Francis Small Teaching Hospital
HCW: Healthcare worker
HIC: High-income country
ICU: Intensive care unit
IV: Intravenous
LBW: Low birthweight
LMIC: Low- and middle-income country
LSHTM: London School of Hygiene and Tropical Medicine
KMC: Kangaroo mother care
MRCG: Medical Research Council The Gambia
NG: Nasogastric
NMR: Neonatal mortality rate
NNU: Neonatal unit
RLS: Resource limited setting
SSNC: Small and sick newborn care
SRQR: Standards for Reporting Qualitative Research
SSA: Sub-Saharan Africa
WASH, Water: sanitation and hygiene
WHO: World Health Organization.
