# Supplementary material for: Barriers and enablers to kangaroo mother care prior to stability from perspectives of Gambian health workers: A qualitative study
Source: Front Pediatr. 2022 Aug 26;10:966904. doi: 10.3389/fped.2022.966904 (PMC9459153; doi:10.3389/fped.2022.966904)
Supplement: Supplementary file 1 [file Data_Sheet_1.pdf]

Health care worker perceptions on early skin-to-skin contact in unstable neonates <2000g in The Gambia

Interview Guide for health-care worker interviews

General principles for interview

- Conduct the interview in a private room and provide refreshments
- Take written informed consent before participation
- Take written informed consent to audio-record the interview

A. Warm-up questions / background of participant

1. What is your role at the hospital and in the neonatal unit?

   (Probe: What's your qualification/education? Describe briefly your daily work.)

2. How long have you worked with neonates?
3. Do you have experience of caring for a preterm/LBW babies?

   (Probe: When? What happened? What's the difference from looking after a term baby? Do you have previous training on this?)

B. Kangaroo mother care

1. Have you ever received training on KMC?

   (Probe: When and where? By whom?)

2. Do you have experience of caring for a preterm/LBW babies who are receiving KMC?

   (*Probe: Who is the provider? Who gives advice to the provider?*

   What are the effects of giving KMC on the baby and the mother/parents? Why does KMC help babies?

   What's your feeling about that?)

3. When do you think KMC should be given to babies?

   (Probe: At what age/weight? Why?

   For how long or at least how long? Why?

   Are there any times when KMC should not be given to preterm/LBW babies?)

4. What do you think about giving babies KMC for 18h/day or longer?

   (Probe: Agree or not? Why? What makes it difficult?)

   - What can you do as a neonatal HCW to promote KMC for longer?

5. What do you think mothers/family members think about giving KMC?

   (Probe: What do they think about giving birth to a preterm/LBW babies?

   Do they think that giving KMC is beneficial? Why?)

6. When a mother goes home, does she continue giving KMC to the baby?

   (Probe: What do you think are the reasons?)

7. What other factors would affect a baby having KMC?
   - What else can you do as a neonatal HCW to make it easier for a baby to receive KMC?

C. Early kangaroo mother care

1. What do you think about giving KMC with other medical treatments, such as oxygen or IV fluids etc.?

   (Probe: What are the difficulties and what are your concerns? How does it work? How does the treatment affect the baby receiving KMC, eg. during ward round or nursing care?)
2. What do you think about starting KMC within the first 24h after delivery?

   (Probe: What are the benefits and difficulties? How would make it easier?)
3. What do you think mothers/care-providers think about doing early KMC at same time as other treatments?
   - What do mothers/care-providers need to provide early KMC?

     (Probe: How to make it easier? How would antenatal education prepare them? How would peer and staff counseling help?)
4. What do you think fathers/other family members think about early KMC?
5. What do you think are the effects of giving early KMC?
6. Is there anything you can do, as a neonatal HCW, to make providing early KMC easier?
7. Is there anything the hospital can do to make providing early KMC easier?

   (Probe: How does the security policy and environment of the hospital affect the early KMC? What resources the hospital provides can be helpful?)
8. Do you have any suggestions to help introduce early KMC at your unit or other units thinking of using it?

D. eKMC trial (Jump here when the interviewee mentions it spontaneously)

1. Are you aware of the eKMC study?
2. Have you received training on eKMC project?

   (Probe: What trainings have you received?)
3. What do you think about this project?

   (Probe: How much do you know it? How differently does it make you think about this intervention? Are you comfortable with it?)
4. Is there anything about the eKMC study that is affecting your ability to care for preterm babies? (either positive or negative effects)

   (Probe: How does it affect? Does it change the way you give advice to mothers/care-ders?)
